# Effect of Dried Mealworms (*Tenebrio molitor*), Larvae and Olive Leaves (*Olea europaea* L.) on Growth Performance, Carcass Yield and Some Blood Parameters of Japanese Quail (*Coturnix coturnix japonica*)

**DOI:** 10.3390/ani11061631

**Published:** 2021-05-31

**Authors:** Asma Ait-Kaki, Jean-Luc Hornick, Samira El Otmani, Youssef Chebli, Nassim Moula

**Affiliations:** 1 Department of Biology, Faculty of Sciences, M’HamedBougara University of Boumerdes, Boumerdes 3500, Algeria; askaki.biotechno@gmail.com; 2 Department of Animal Production, Fundamental and Applied Research for Animal and Health, Faculty of Veterinary Medicine, Liege University, B-4000 Liege, Belgium; jlhornick@uliege.be; 3 National Institute of Agricultural Research (INRA), 78 Bd. Mohamed Ben Abdellah, Tangier 90010, Morocco; samira.elotmani@inra.ma (S.E.O.); youssef.chebli@inra.ma (Y.C.); 4 GIGA Animal Facilities, Liege University, B-4000 Liege, Belgium

**Keywords:** quail, *Tenebrio molitor*, olive leaf, animal performance, carcass, serum constituents

## Abstract

**Simple Summary:**

Soybean is the most important protein source in poultry feed. Due to its high importation cost and its production arising generally from genetically modified organisms (GMOs), insects are currently considered to be a low-cost and eco-friendly alternative. In the present work, the inclusion of *Tenebrio molitor* (TM) insect meal and/or olive leaves (OL) in the basal quail diet has been tested on growth performance, carcass yield and some blood parameters. The results showed that 3% inclusion TM and 2% inclusion OL improved quail body weight at 5 weeks old, reduced FCR and did not negatively influence carcass yield and blood parameters of Japanese quail. In conclusion, the present work can contribute to a strategy aimed at reducing the use of conventional poultry feed ingredients, which might reduce the feeding costs of quails in Algeria.

**Abstract:**

The aim of this study was to investigate the effect of *Tenebrio molitor* meal (TM) and/or olive leaf powder (OL) supplementation to quail diet on animal performance, carcass yield and some blood parameters. One hundred and forty-four 1-day-old Japanese quails (body weight: 29.9 ± 0.46 g) were divided into four groups of 36 chicks, receiving four different diets, i.e., G1: Standard commercial (SCD); G2: SCD + 3% TM; G3: SCD + 3% OL; and G4: SCD + 3% TM + 2% OL. Feed and water were provided *ad-libitum*. The results showed that TM and OL inclusion promoted quail body weight at 5 weeks of age; *p* = 0.001. Feed conversion ratio (FCR) of G3 was significantly (*p* < 0.01) reduced compared to the other groups. Overall mortality was not different, at around 6.25%, between groups. Carcass [(71.5–74.3%)], gizzard [(2.5–2.7%)], heart [(1.1–1.2%)] and giblet [(6.8–6.9%)] yields were not influenced by the diet. No significant effect of the diet was observed for serum proteins, creatinine, urea and lipids. To conclude, the mixture of an incorporation of TM and OL in quail diet showed no adverse effects on growth performance, carcass constituent yields and serum constituents.

## 1. Introduction

Soybean is considered to be a core of the modern animal feed system. However, the high importation costs of soybean and its production from genetically modified organisms (GMOs) have added additional constraints to poultry production worldwide [1]. Therefore, it is worth identifying safe, locally available and low-cost protein sources to replace soybean. Insect meals have been addressed as possible feed alternatives to soya, because of their rich nutrient content and extremely low environmental impact [2]. In Algeria, soya, widely used in poultry feed, is imported from abroad, which has a negative impact on the production cost of poultry meat. The use of locally produced insects could reduce the costs of animal production.

Olive leaves have been used in animal feed for their health benefits such as hypotensive [3], hypoglycemic, anti-oxidant [4], and anti-infectious effects [5]. Hence, olive leaf supplementation should be used at medium levels for better animal health and production.

Thus, the present work aimed to investigate the effects of dried *Tenebrio molitor* (TM) larvae and/or olive (*Olea europaea*) leaf powder (OL) incorporation in the quail diet on animal performance, carcass yield, and some serum parameters.

## 2. Materials and Methods

### 2.1. Experimental Site 

The experiment was conducted in a poultry farm of Bejaia province (06770), Algeria.

### 2.2. Animals and Housing

The experience was carried in 2020 for 5 weeks (from 31 March to 8 May). One hundred and forty-four 1-day-old Japanese quail chicks (body weight: 29.9 ± 0.46 g) were used in this study. They were randomly distributed into four groups of 36 chicks each, with 3 repetitions of 12 animals per group, according to the diet used. Growing and finishing diets were formulated. Quail chicks in Group 1 were fed a control feed (Diet 1); Group 2, Group 3 and Group 4 received the Diet 1 substituted with, respectively, 3% dried *Tenebrio molitor* (TM) larvae (Diet 2), 3% olive leaves powder (Diet 3); and 3% TM plus 2% of OL (Diet 4). Feed and water were provided ad-libitum. The formulation of the basic diet was adopted according to the feed marketed in Algeria for quails. Composition of the feed is given in Table 1.

A total of 12 boxes, 100 × 80 × 200 cm, were used to house the animals, with twelve quails per box, according to a completely random block design (Table 2). Quail Chicks received daily 16 h light/8 h dark. The temperature was maintained between 38 and 40 °C until the end of the second week when it was lowered to 34–35 °C by reducing the number of heaters. After the 25th day of experiment, the room temperature was maintained at 20 °C. 

### 2.3. Data Collection

The amounts of feed distributed and refused in the feeders were measured daily. Animals were individually weighed at days 0, 7, 21 and 35 of the experiment. The feed conversion ratio (FCR) was calculated at the end of the experiment as the ratio of the amount of feed ingested during the rearing period to total weight gain. At the end of the experiment, the quails were fed for about 12 h and the animals were then slaughtered. They were manually plucked and 1 h after slaughter, and the carcass weight was calculated by removing feathers, blood, head, tarsus and organs. Carcass yield was measured as a percentage of live weight. Liver, gizzard, heart, giblets and dressing yields were obtained as a percentage of carcass weight. At the end of the experiment, all quails were stunned before slaughter. The quails were then suspended alive from a mobile metal stand that held them by the legs, head down. The quails were then shot by cutting the jugular vein with a sharp knife and were left hanging until the bleeding stopped. At the same time, approximately 3 mL of blood was collected from three randomly selected quails in each pen in red-top tubes without anticoagulant for serum biochemical analysis. The samples were transported to the laboratory on the same day.

### 2.4. Biochemical Parameters

Biochemical parameters were analyzed with the “Biosystems BA20” automaton (Biosystems S.A., Barcelona, Spain). The parameters measured were total proteins, albumin, globulin, creatinine, urea, total cholesterol, triglycerides, high-density lipoprotein (HDL), low-density lipoprotein (LDL), very low-density lipoprotein (VLDL). 

### 2.5. Statistical Analyses

All statistical analyses were performed using the Statistical Analysis System (SAS) 9.2 software (Cary, NC, USA). An analysis of variance was used to study the effects of diet on the different parameters studied.

## 3. Results and Discussion

The overall mortality was not significantly affected by the diet, at around 6.25% (*p* > 0.05) for all tested groups (Table 3). The use of TM in the diet of group 2 (TM (3%)) significantly (*p* < 0.05) decreased the body weight (BW) of the birds at the age of 5 weeks compared to group 1 (178.9 vs. 190), while OL supplementation (OL (3%)) had no significant effect on this parameter. However, the inclusion of both TM and OL in (TM (3%) + OL (2%)) increased significantly the BW of the birds (205 vs. <192 g; *p* = 0.001). The feed conversion ratio was significantly reduced in group 3 (*p* < 0.01: 2.78 vs. >3) (Table 3).

Zadeh et al. [6] and Jabri et al. [7] reported, respectively, the positive effects of TM meal and olive leaf supplementation in chicken’s diet. Herbal extracts have been reported to improve animal performances by regulating the digestion activity, stimulating appetite and feed intake, and having antibacterial effects [8]. These effects are affected by the plant species incorporated in animal diets, as well as the type of extracts (herbal extract, essential oils, fresh plant, dried plants, etc.), and their quantities [9,10]. However, adverse effects on growth performances of chicken were recorded, with diets containing 50 to 100 g TM/kg diet [11,12]. 

In the present work, carcass (71.5–74.3%), gizzard (2.5–2.7%), heart (1.1–1.2%) and giblet (6.8–6.9%) yields were not significantly (*p* > 0.05) influenced by the diet (Table 4). Zadeh et al. [9] reported similar observations for quails fed with a basal diet supplemented with TM. In contrast, Bovera, et al. [2] observed the highest weight of carcass in broilers fed with TM larvae meal as a protein source due to their high protein quality and quantity [13]. It is noteworthy that the liver yield was significantly higher in the group that received OL alone, when compared to the other groups (Table 4).

No significant effect on the diet was observed for serum constituents, as shown in Table 5. Similar results were found for serum glucose, cholesterol and triglyceride in the hens fed with vitamin E and olive leaf extracts [14]. In contrast, serum cholesterol, glucose and triglycerides levels were significantly decreased, with increasing levels of olive leaf extract in quail’s drinking water [15].

## 4. Conclusions

In conclusion, partial substitution of a conventional diet with 3% of dried *Tenebrio molitor* larvae, or 2% of *Olea europaea* leaf powder in the diet gave inconsistent positive results on body weight at 5 weeks old and feed conversion ratio and did not influence the carcass yield or blood parameters of Japanese quail. 

## Figures and Tables

**Table 1:** Feed composition of control feed (growing and finishing diets) offered to Japanese quail, and composition of *Tenebrio molitor* or olive leaves powder.

Ingredients (%)	Grower				Finisher			
	SCD	SCD + OL3%	SCD + TM 3%	TM (3%) + OL (2%)	SCD	SCD + OL3%	SCD + TM 3%	TM (3%) + OL (2%)
Corn	55	53.35	53.35	52.25	62	60.14	60.14	58.9
Soybean meal	36	34.92	34.92	34.2	23	22.31	22.31	21.85
Bran	5	4.85	4.85	4.75	12	11.64	11.64	11.4
Limestone	1.8	1.75	1.75	1.71	1.2	1.16	1.16	1.14
Bicalcium Phosphate	1.2	1.16	1.16	1.14	0.8	0.776	0.776	0.76
Multivitamin and Mineral Complex	1	0.97	0.97	0.95	1	0.97	0.97	0.95
OL	0	3	0	2	0	3	0	2
TM	0	0	3	3	0	0	3	3
**Chemical Composition**								
Dry Matter (%)	87.1	86.06	87.33	86.63	86.8	84.21	87.03	85.31
Metabolic Energy (kcal/kg)	2631	2608	2689	2674	2688	2663	2744	2728
Crude Protein (%)	21.2	20.92	22.15	21.7	17	16.84	18.08	17.83
Calcium (%)	0.8	0.83	0.78	0.83	0.76	0.79	0.74	0.8
Lysine (%)	1.52	1.47	1.65	1.58	1.22	1.18	1.36	1.3
Methionine (%)	0.8	0.78	0.82	0.81	0.74	0.72	0.76	0.75

Grower: 0 to 3rd week; Finisher 4th to 5th week; SCD: Standard Commercialized Diet; OL: Olive Leaves; TM: *Tenebrio molitor.*

**Table 2:** Random assignment of groups.

Block1		Block2
TM (3%) + OL (2%)	Corridor	TM (3%)
Control		TM (3%) + OL (2%)
Control		OL (3%)
TM (3%)		OL (3%)
TM(3%)		OL (3%)
TM (3%) + OL (2%)		Control

OL: Olive Leaves; TM: Tenebrio molitor.

**Table 3:** Effect of *Tenebrio molitor* (TM) meal and/or olive leaf (OL) powder on quail performance.

Title Item	Control	TM (3%)	OL (3%)	TM (3%) + OL (2%)	SEM	*p*-Value
Week	Live Weight (g)					
0	11.86	11.77	11.82	11.78	0.09	0.88
1	29.96	29.81	29.75	30.01	0.12	0.46
3	103.96 ^ab^	101.74 ^b^	107.48 ^ab^	108.79 ^a^	2.13	0.09
5	189.99 ^a^	178.9 ^c^	191.93 ^a^	204.96 ^b^	2.93	<0.001
	**ADG (g/day)**					
0–3	4.39	4.28	4.56	4.62	-	-
4–5	6.15	5.51	6.03	6.87	-	-
0–5	5.09	4.78	5.15	5.52	-	-
	**Feed Intake (g/day)**					
0–5	15.32	14.56	14.32	16.72	-	-
	**Feed Conversion Ratio**					
1–5	3.01 ^a^	3.05 ^a^	2.78 ^b^	3.03 ^a^	0.06	0.01

ADG: average daily gain; *p*: probability; OL: Olive Leaves; TM: *Tenebrio molitor*. SEM: standard error of the mean. Means with different capital letters (a–c) in the same row indicate significant differences (*p* < 0.05).

**Table 4:** Effect of *Tenebrio molitor* (TM) meal and/or olive leaf (OL) powder on carcass and organ yields.

Item	Control	TM (3%)	OL (3%)	Control	TM (3%)	OL (3%)
Carcass %	71.53 ± 1.06	74.25 ± 0.79	72.17 ± 0.93	73.61 ± 0.74	0.89	0.12
Liver %	3.27 ^a^ ± 0.04	3.25 ^a^ ± 0.04	3.49 ^b^ ± 0.03	3.32 ^a^ ± 0.05	0.04	0.01
Gizzard %	2.65 ± 0.04	2.53 ± 0.04	2.62 ± 0.04	2.61 ± 0.04	0.05	0.22
Heart %	1.19 ± 0.04	1.11 ± 0.04	1.1 ± 0.04	1.13 ± 0.04	0.04	0.48
Giblets %	6.82 ± 0.03	6.85 ± 0.03	6.84 ± 0.03	6.86 ± 0.03	0.03	0.82

OL: Olive Leaves; TM: *Tenebrio molitor*; SEM: standard error of the mean. Means with different capital letters ^(a,b)^ in the same row indicate significant differences (*p* < 0.05).

**Table 5:** Effect of dietary treatments on blood constituents and lipid profile of Japanese quail serum.

Item	Control	TM (3%)	OL (3%)	TM (3%) + OL (2%)	SEM	*p*-Value
TP (g/dL)	3.02 ± 0.07	3.04 ± 0.05	2.97 ± 0.08	3.15 ± 0.07	0.07	0.34
Alb (g/dL)	1.30 ± 0.02	1.35 ± 0.03	1.33 ± 0.08	1.31 ± 0.07	0.02	0.46
Glob (g/dL)	1.70 ± 0.04	1.68 ± 0.04	1.72 ± 0.04	1.71 ± 0.03	0.04	0.97
A/G (%)	0.77 ± 0.02	0.80 ± 0.02	0.78 ± 0.03	0.76 ± 0.02	0.01	0.71
Creatinine (mg/dL)	0.27 ± 0.01	0.25 ± 0.01	0.28 ± 0.01	0.26 ± 0.01	0.01	0.34
Urea (g/dL)	6.81 ± 0.13	6.67 ± 0.12	6.76 ± 0.13	6.53 ± 0.14	0.13	0.46
TG (mg/dL)	218.5 ± 6.51	220.17 ± 6.83	220.08 ± 8.65	214.58 ± 4.84	6.85	0.93
HDL (mg/dL)	54.5 ± 1.57	56.05 ± 1.69	54.33 ± 1.55	55.75 ± 2.05	1.73	0.87
LDL (mg/dL)	95.83 ± 2.64	92.67 ± 1.78	91.58 ± 2.70	99.08 ± 2.16	2.35	0.12
VLDL (mg/dL)	43.58 ± 1.57	42.41 ± 1.26	43.75 ± 1.71	45.08 ± 1.59	1.52	0.68
TC (mg/dL)	190.67 ± 2.59	196.83 ± 2.61	194.33 ± 2.60	197.01 ± 3.05	2.72	0.33

TP: Total Proteins; Alb: Albumin; Glob: Globulin; A/G: Alb/Glob ratio; TG: Triglycerids; TC: Total Cholesterol; TM: *Tenebrio molitor*; OL: olive leaves; *p*: probability; SEM: standard error of the mean.

## Data Availability

The data presented in this study are available from the corresponding author on request.
